# Mammary analogue secretory carcinoma in minor salivary gland

**DOI:** 10.31744/einstein_journal/2022RC5724

**Published:** 2022-02-08

**Authors:** Lucas Moreno Ponso, Victor Pereira Zerbinatti, Sílvia Miguéis Picado Petrarolha, Vania Loureiro, Rogério Aparecido Dedivitis

**Affiliations:** 1 Universidade Metropolitana de Santos Santos SP Brazil Universidade Metropolitana de Santos, Santos, SP, Brazil.

**Keywords:** Carcinoma, Salivary gland neoplasms, Salivary glands, minor, Mammary analogue secretory carcinoma

## Abstract

Mammary analogue secretory carcinoma is a rare neoplasm usually confused with other neoplasms in the salivary glands region. It has great similarity with the breast carcinoma. We report a case of a patient who presented with gingival submucosal bleeding and lesion, with the initial histopathological examination revealing salivary gland neoplasm of low crane. Computed tomography revealed the lesion near the tooth 27, with extension to the floor of the left maxillary sinus and to the palate mucosa. Resection of the infra-structure was performed, with a diagnosis of breast cancer secretory carcinoma in the minor salivary gland.

## INTRODUCTION

Mammary analog secretory carcinoma (MASC) is a rare salivary gland tumor that was first described by Skálová et al.,^(1)^ It has the same histological and molecular composition as secretory breast carcinoma,^(2)^ without granular zymogens and a positive result for mammaglobin, and colloid-like extracellular material. These features differentiate it from a typical salivary gland tumor.^(1)^ Mammary analog secretory carcinoma is quite rare. From its first report in 2010 until 2016, 90 cases have been documented. Of these, two-thirds have been described in parotid glands and the remainder in minor salivary and submandibular glands.^(3)^

Mammary analog secretory carcinoma shares features of ETV6-NTRK3 protein translocation and immunophenotype with secretory breast carcinoma. This fused gene encodes an atypical tyrosine kinase enzyme, which has a transforming potential that plays a major role in oncogenesis. A molecular tyrosine kinase inhibitor can be used as a treatment for patients carrying the ETV6-NTRK3 gene.^(1)^

Previous studies have diagnosed MASC as low-grade acinar cell carcinoma, in which a painless, slow-growing mass develops with a generally favorable prognosis. However, there are reported cases of more aggressive forms of MASC, in which lymph node metastasis and even deaths related to this carcinoma have occurred.^(4)^

## CASE REPORT

**Figure 1:**
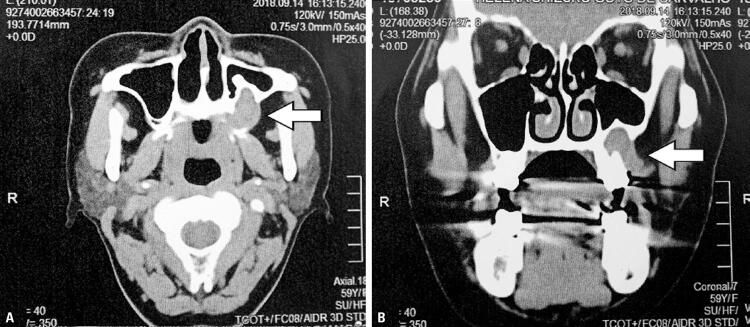
Computed tomography in axial (A) and coronal (B) slices, showing a mass with little enhancement in the left pterygopalatine fossa (white arrows) that was pushing ventrally to the posterior wall of the ipsilateral maxillary sinus, without evidence of bone lysis

**Figure 2:**
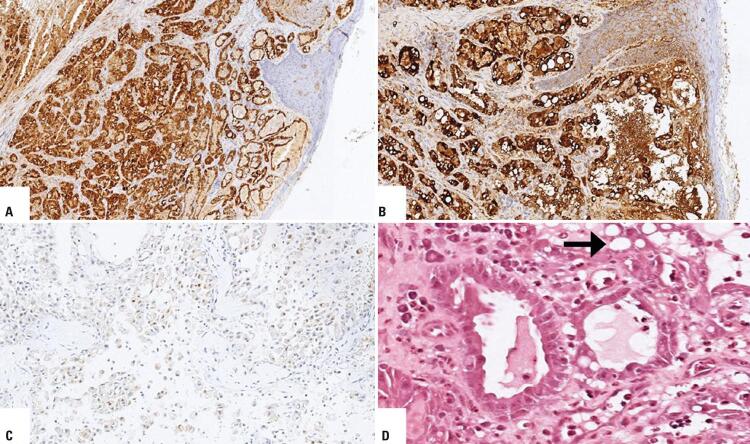
Immunohistochemistry showing labeling profile with (A) S100+ protein, (B) mammoglobin+, discovered on GIST-1 - SRY-related HMg-BOX gene 10- and (C) neurotrophic tyrosine receptor kinase+, in addition to (D) obvious acinar differentiation (numerous zymogen granules with diffuse positivity - arrow)

A 56-year-old woman who reported slight gingival bleeding for 3 months, lasting for 5 days, which spontaneously stopped. She was evaluated by a dentist, who detected a gingival lesion and performed a biopsy for histopathological examination. She denied smoking and consume alcohol and she reported used eye drops for glaucoma. The histopathological examination revealed low-grade epithelial neoplasia that was suggestive of low-grade acinar cell carcinoma of the minor salivary gland. Upon admission, she was in good general condition, and locoregional examination showed a smooth-surfaced nodular lesion of about 8x3mm near the second upper left molar (tooth 27), with no evidence of lymph node enlargement. Computed tomography revealed a 25x22x16mm lesion near tooth 27, extending to the floor of the left maxillary sinus, with extension to the palate mucosa (Figure 1). She underwent infrastructure resection (partial maxillectomy by osteotomies) with intraoperative exodontia, without cervical emptying, and with reconstruction using an advancing jugal flap. The surgical margins came free - confirming the intraoperative freezing examination. The bone tissue was free of neoplastic involvement. The patient had a good progress. The histopathological diagnosis showed MASC (Figure 2). The patient continued under outpatient follow-up, with no clinical or radiological evidence of recurrence after 10 months.

This study was approved by the Research Ethics Committee of *Universidade Metropolitana de Santos* under number #3.534.532, CAAE: 09615219.5.0000.5509.

## DISCUSSION

Mammary analog secretory carcinoma is a rare tumor with about 90 cases reported in the published literature. This neoplasm was not recognized as a distinct entity until 2010, for this reason, this is not listed in the 2005 World Health Organization (WHO) classification of salivary gland tumors.^(3)^ It is important to have histopathological knowledge of this disease, to differentiate it from other salivary gland lesions, such as low-grade acinar cell carcinoma (the main differential diagnosis),^(5)^ adenocarcinoma cyst, and low-grade mucoepidermoid carcinoma.^(1)^ The clinical behavior of MASC is still unknown, with rare cases of recurrence and aggressive profile. The correct recognition provides better observation of its biological behavior pattern, which can stratify candidate cases for adjuvant treatment.^(5)^

However, there are differences in the immunohistochemical profile: mammary cell carcinoma reacts with mammaglobin and S100 protein, while acinar cell carcinoma does not show appreciable expression of these markers. The morphology of these two tumors may be similar, which can lead to misinterpretation.^(3)^

The case we report was initially diagnosed as low-grade acinar cell carcinoma at the time that the biopsy was performed. It was classified as MASC only after histopathological confirmation of the surgical specimen. The tumor growth site is also consistent with finding reported in the published literature. The lesion extended to the palate mucosa, which is characteristic of MASC,^(3)^ and size of the nodule was approximately 24mm in size.^(1)^ It may share microscopic similarities with other neoplasms, and its final diagnosis was based on the presence of the molecular alteration, which is the fusion of the ETV6-NTRK3 gene.^(1)^

The most common site of origin of MASC is the parotid gland. However, occasionally it can affect the submandibular or minor salivary gland of the lips, soft palate, and jugal region. Although cytopathological diagnosis is difficult (as in our case), the determination of malignancy is important, for decision making in favoring of surgical treatment.^(4)^ Immunohistochemical study is necessary for diagnostic confirmation, preferably supported by molecular studies.^(2)^

The clinical behavior of MASC is still not well known. Due to the small number of cases described, information on long-term follow-up is still limited. It is considered a low-grade carcinoma with favorable prognosis. However, rare cases have also presented relapse, lymph node metastasis, and even death related to the disease.^(5)^
